# Description of Behavioral Patterns Displayed by a Recently Weaned Cohort of Healthy Dairy Calves

**DOI:** 10.3390/ani10122452

**Published:** 2020-12-21

**Authors:** John Alawneh, Michelle Barreto, Kealeboga Bome, Martin Soust

**Affiliations:** 1 Good Clinical Practice Research Group (GCPRG), School of Veterinary Science, The University of Queensland, Gatton, QLD 4343, Australia; m.barreto@uq.net.au (M.B.); MartinS@terragen.com.au (M.S.); 2 School of Veterinary Science, The University of Queensland, Gatton, QLD 4343, Australia; kbime76@gmail.com; 3 Terragen Biotech Pty Ltd., Coolum Beach, QLD 4573, Australia

**Keywords:** calves, movement patterns, GPS, health, welfare

## Abstract

**Simple Summary:**

Modern technology has allowed researchers to track the movement patterns of cattle with increasing accuracy in order to gain a greater understanding of both overt and subtle activity trends. The aim of this study was to describe and analyze movement patterns displayed by recently weaned and healthy dairy calves. Three movement pattern clusters were identified, and calves in this study were more active in the afternoon and at night. There was a correlation between the rate of movement, linearity ratio, and the distance traveled. However, turning angles do not have any influence on the distance traveled and the rate of movement across the three cluster-type movements. The findings reported in this study could be used to further develop the interpretation of movement and behavior patterns of calves in order to establish an early detection system for poor health and welfare on dairy farms.

**Abstract:**

Animals display movement patterns that can be used as health indicators. The movement of dairy cattle can be characterized into three distinct cluster types. These are cluster type 1 (resting), cluster type 2 (traveling), and cluster type 3 (searching). This study aimed to analyze the movement patterns of healthy calves and assess the relationship between the variables that constitute the three cluster types. Eleven Holstein calves were fitted with GPS data loggers, which recorded their movement over a two week period during spring. The GPS data loggers captured longitude and latitude coordinates, distance, time and speed. It was found that the calves were most active during the afternoon and at night. Slight inconsistencies from previous studies were found in the cluster movements. Cluster type 2 (traveling) reported the fastest rate of movement, whereas cluster type 1 (resting) reported the slowest. These diverse movement patterns could be used to enhance the assessment of dairy animal health and welfare on farms.

## 1. Introduction

Animals display distinct movement patterns and behaviors that can be monitored over time and used as a health indicator [1]. Modern technology has provided new avenues that facilitate study into the movement patterns of livestock [2,3]. Such research has coincided with the ongoing development of precision livestock farming (PLF) techniques as a means to survey livestock wellbeing continuously and more effectively [4]. PLF has adopted smart farming techniques to improve the sustainability of animal production, and animal monitoring is both ethically and economically beneficial in this goal [5,6]. Global positioning system (GPS) tracking, in particular, has been implemented as a tool for using location data to assess livestock behavior [7,8,9]. GPS technology in cattle has also been used to study the environmental impacts of grazing behavior [10] and feeding site selection [11]. Such studies have offered refinements to available GPS technology and the interpretation of behavior patterns [12].

Health problems typically result in loss of production, high treatment costs and can have devastating impacts on the welfare of cattle [13]. Conditions such as mastitis and lameness are common in dairy cattle with serious economic impacts [14,15,16]. These disorders result in behaviors associated with pain and discomfort, such as abnormal lying periods and reduced feed intake [17,18]. Previous studies have reported that behavior is the first thing to be affected by a disease state [19]. In calves, changes in activity levels in association with disease state have been monitored [20,21,22]. Therefore, accurate real-time monitoring of behavior could be used as an early disease detection tool and subsequently reduce the time taken to treat sick animals [17]. Early diagnosis and treatment of disease could minimize economic losses that typically result from decreased productivity and high mortality rates [5]. In addition to early signs of disease, behavioral changes may also indicate physiological processes such as estrus and can, therefore, also be used in routine reproductive management [23,24,25]. However, the challenge for farm owners has always been how to accurately monitor these behaviors [26].

Dairy farmers have traditionally relied on farmworker observations to identify unhealthy animals [23,26,27]. However, the limitations of direct human observation are unpredictable accuracy, high time consumption and impracticality on large-scale commercial farms [28]. Furthermore, cattle tend to mask sickness by altering their behavior to avoid recognition, especially in the early stages of the disease [12]. As a result, subclinical diseases or conditions, in particular, may escape farmworker detection due to the absence of clinical signs [29]. Technology-based automatic tracking of animal behavior minimizes the risk of human error and can be used for extended periods when compared to direct human observation [30]. A previous study has demonstrated the efficiency of using data-loggers to monitor animal behavior and interpret the results [31].

GPS technology has allowed researchers to track the movement patterns of cattle with increasing accuracy to gain a greater understanding of both overt and subtle activity trends [32]. Movement patterns may be in the form of walking or running, turns, or simply lying down. The movement patterns of cattle provide information on not only the performance of activities such as feeding, drinking or lying down but also on time spent in each of these activities [33]. For example, O’Driscoll et al. [34] tracked the standing and lying behavior of dairy cows using data-loggers and reported a link between lying patterns and feed availability. This indicates that feed restriction or nutritional deficiency can result in subtle changes such as patterns of lying behavior. Such spatiotemporal information is useful in dairy farming where feed intake influences milk production, and prolonged lying may be a sign of lameness [27,29,35]. Movement patterns can also be used in tracking sources of infections and animals that might have been in contact with the infected ones [36,37].

Algorithms of behavior that can be used to identify the presence of a health condition, such as lameness or mastitis, have already been developed in mature dairy cows [3]. However, to the authors’ knowledge, there have not been studies conducted that aimed to quantify the normal movement patterns of healthy dairy calves by use of GPS tracking. Previous studies measuring the activity of calves have typically used accelerometers alone [38,39,40,41]. Accelerometers use body acceleration across up to three spatial axes to provide information on body position and activity patterns [42]. GPS data, on the other hand, provide information on the location, through longitude and latitude coordinates, of animals and the time spent in these locations to deduce movement patterns [43]. Both options are relatively inexpensive and can be used to monitor animal behavior and movement patterns either alone or in combination with each other; however, GPS data are more useful when location information and the quantification of traveling distance is desired [2]. Therefore, this study aimed to describe and analyze the diversity of the movement patterns displayed by a recently weaned cohort of healthy dairy calves using GPS tracking. In doing so, this study also aimed to develop a dataset of movements characteristic of healthy calves.

## 2. Materials and Methods

The study was carried out following approval from the University of Queensland Animal Welfare and Ethics Committee (Approval number: SVS/064/15/ARC).

### 2.1. Animal Selection and Housing

This pilot cohort study was conducted on dairy calves at the University of Queensland, Gatton campus (27.552297S, 152.333471E), in southeast Queensland during September 2015 (Average temperature 15 °C, range = 9–21 °C, average rainfall = 26 mm). Eleven Holstein calves of different sexes (males: *n* = 6, females: *n* = 5) and aged 8–9 weeks were enrolled in the study. Each calf was earmarked with a unique visual identification number. The calves were managed as one group in an open fenced grazing (ryegrass) paddock, which measured approximately 105 m × 30 m. The study calves had ad libitum access to feed and clean water and access to a shade structure (3 m × 6 m × 2 m) located on the eastern side of the paddock. The calves ration was made up of hay and concentrates of pellets (Calf Starter Crumble, Norco^®^, Clifton Grove, NSW, Australia). In order to further limit the disruption to the behavior of the calves, the feed was delivered once at the start of the study period via tractors and placed into a grain self-feeder (Paton™ one ton self-feeder, Paton Industries, Victoria, Australia). The feeder was placed in the center of the claves paddock within 10 m of the water source. The movement of the calves within the paddock was unrestricted.

### 2.2. Data Collection and Management

The calves were fitted with waterproof geographic positioning systems (GPS) data loggers (Igot-U GT600, Mobile Action Technology Inc., Taipei, Taiwan) to capture coordinates (longitudes and latitudes), distance (m), time and speed (m/s) for two weeks. The loggers themselves measured 46 mm × 41.5 mm × 14 mm and weighing 37 g. The ear tag of each calf and the serial number of the GPS logger unit were linked together. The combination of ear tag and GPS unit serial number was used as a form of identification during the study.

The GPS loggers were placed into collar pouches and fitted around the calves’ necks. Fitting GPS loggers around the neck ensures that they can effectively access satellite signals without much interference [44]. The GT600 could capture 2668 points over two weeks as it has a power-saving mode [45]. The data loggers were preset to record data at twenty-second intervals over a twenty-four-hour period for a duration of two weeks. A twenty-second interval reduced the chances of omitting little movements [44]. Mobile action technology software @trip (Mobile Action Technology Inc., Taipei, Taiwan) was used to import data collected. The accuracy of the positions captured was shown to be 4.4 m [44]. Animal behaviors were not recorded in this pilot study in an attempt to minimize disturbances to normal calves’ behavioral patterns. The data were downloaded from the GPS data loggers using the @trip software by connecting the device to a computer using a USB cable. The data were then saved as *.gpx files and converted to *.csv files and included information such as the date and time that the data were captured.

### 2.3. Movement Analysis

The following variables were calculated to describe the movement patterns of the calves; the number of relocations (i.e., moves; count), distance (m) between moves, rate of movement (ROM; m/s), angles of turns (i.e., changes in direction between movements), and linearity ratio. ROM was calculated as the distance between each pair of successive relocations divided by the second [46]. Turning angles were derived from the change of the coordinates and then expressed in radians. The linearity ratio is used to indicate whether the movement is in a straight line (direct) or not [33,46]. Linearity ratio was obtained by measuring the linear distance between two sets of movement and dividing by the total distance traveled by a calf. Therefore, ROM values closer to one indicate linear movement while those leaning towards zero signify turning movements [46].

The mean and standard deviation were calculated for all the variables. The correlation between the variables was quantified using Spearman’s correlation coefficient (rho) to describe the relationship between them. The correlation and correlation matrix plot was generated using the R statistical package corrplot [47]. Agglomerative hierarchical [48,49] and K-means cluster analyses were used to identify similar subgroups of movement patterns “homogenously” clustered based on distance, relocations, ROM, linearity scores and turning angles. Using an iterative procedure, the agglomerative hierarchical clustering algorithm joined at each stage the two most similar clusters until there was a single cluster. At each stage, squared distances between clusters were recomputed by the Lance–Williams dissimilarity update formula according to Ward’s minimum variance method. Ward’s method was selected because clusters are merged based on the minimum increase in total within-cluster variance after merging, therefore resulting in compact and spherical clusters [48,49]. Similarly, for the K-means clustering, individual calf measurements were initially assigned to their own cluster, therefore, setting the maximum number of clusters in the dataset. Using the Hartigan–Wong algorithm [50], the K-means clustering joined at each stage the two most clusters that minimize the within-cluster sum of squares until there was a single cluster. Validation of the clustering algorithms appropriateness and the number of clusters was carried out by assessing the internal measures connectivity, and silhouette width, and Dunn index for each algorithm. Cluster analyses were conducted using Factorextra [51], clValid [52] and stats packages implemented in R [53]. One-way analyses of variance (ANOVA) with repeated measures within calf (calf was fitted as the error term in the model) were used to test for the significance of distance, rate of movement, linearity ratio, and turning angle variables in characterizing calves movement pattern cluster. Chi-squared test was used to test the homogeneity of calves’ relocations (in counts) between the observed clusters. Analyses were done using the fpc [54] and adehabitat packages [55] implemented in R.

## 3. Results

**Figure 1:**
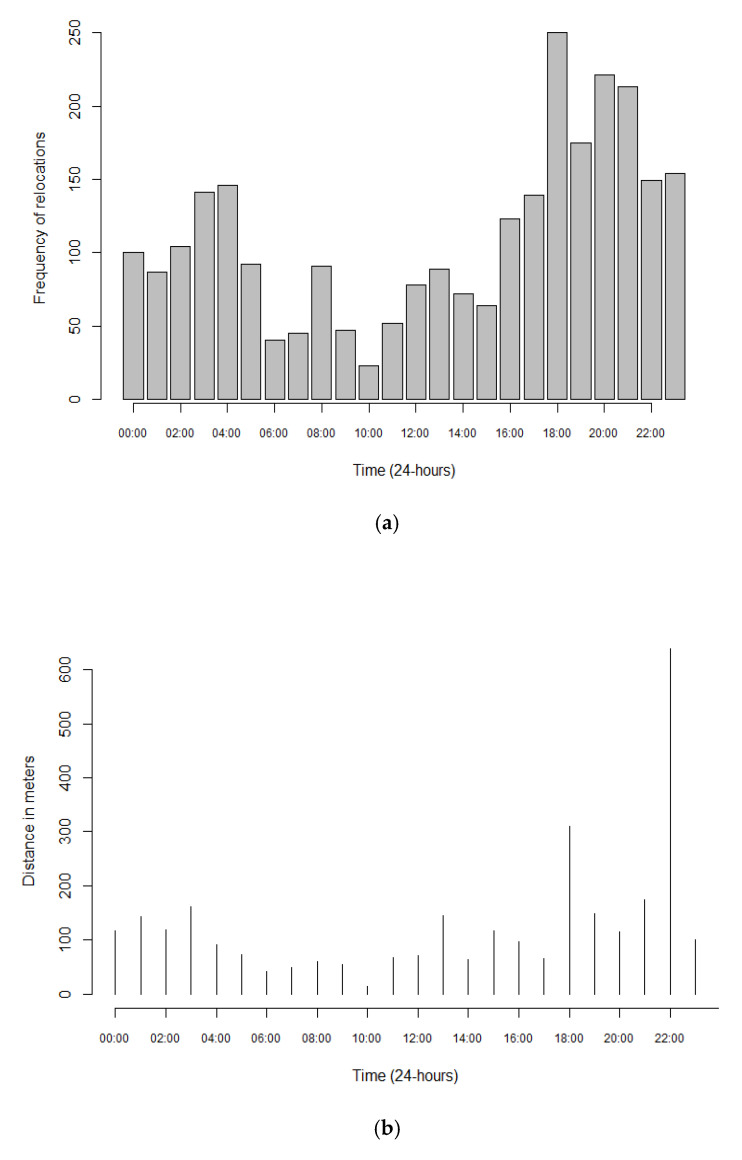
Frequency of relocations (count; (**a**)) and the corresponding distance (m; (**b**)) traveled by the calves over a 24 h period.

Of the 14 calves that were fitted with GPS loggers, three failed to record any data, and therefore these calves were omitted from the results and subsequent analysis. No calves became sick during the study. There were 17,532 relocations made by the 11 calves. There were eight calves that recorded over 1000 relocations each and only three that recorded less than 1000 relocations. The smallest number of relocations made by one individual calf was 618, and the highest number of relocations made was 3725. The average distance per relocation was 18 m traveled at an average speed of 0.67 m/s (Table 1). The highest number of relocations and the greatest distance between relocations were recorded at 6 pm and between 8 and 9 pm (Figure 1a,b).

Calves’ distance traveled, ROM, linearity ratio and the turning angle variables were used to characterize the three cluster types of movement (Table 1). Turning angle was weakly correlated with rate of movement (*rho* = 0.06; *p* < 0.01), linearity ratio (*rho* = 0.09; *p* < 0.01), and distance (*rho* = 0.10; *p* = 0.02). Rate of movement was strongly correlated with linearity ratio (*rho* = 0.67; *p* < 0.01) and distance (*rho* = 0.69; *p* < 0.0), while linearity ratio was strongly correlated with distance (*rho* = 0.91; *p* = 0.01).

**Figure 2:**
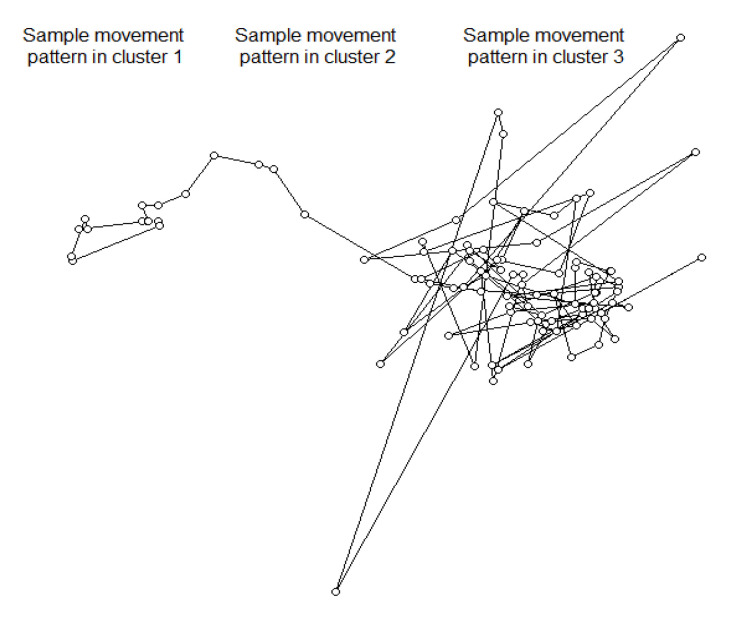
An illustration of the three cluster type movements: cluster 1, resting; cluster 2, traveling; cluster 3, foraging.

Distance, ROM and linearity ratio were associated (*p* < 0.01) with movement pattern clusters. Cluster type 2 movement pattern had the greatest mean distance between moves (Table 1; *p* < 0.01), the highest rate of movement (*p* < 0.01), the highest linearity ratio (*p* < 0.01), and were followed by the same measurements for cluster type 3 and then 1 (Table 1). Turning angle tended to differ between clusters (*p* = 0.09). An illustration of the three movement clusters is displayed in Figure 2.

## 4. Discussion

Animal monitoring has the potential to improve management practices by offering an early detection system that indicates situations of poor welfare, including disease [56]. Precision livestock farming techniques, such as automatic animal tracking, are essential for the economic, environmental and ethical sustainability of livestock agriculture [57]. Although many behavior monitoring techniques have been proposed and tested in the literature, further research into the interpretation of the collected behavioural data is useful for the development of efficient systems that could be employed on farms [5]. This preliminary study provided a dataset of distinct movement patterns displayed by healthy Holstein calves over two weeks during the spring season. These data could represent the normal and expected movement patterns of healthy Holstein calves. A practical application of these data could be that it may be incorporated into the assessment of GPS tracking data of a similar cohort of calves on farms, where deviations from these expected movement patterns may trigger a health alert. For example, if a calf exhibits low levels of activity during a period of expected high activity, then such a calf could be subclinically physiologically or pathologically compromised and should be investigated further. Ultimately, these findings could be used to build an early detection system for the production of animal diseases.

Three distinct movement pattern clusters were identified in the current study based on relocation and movement data. Our findings are compatible with previously described movement patterns for mature cattle [58,59]. The first category is the resting or bedding movement pattern (cluster Type 1). The second pattern is the traveling mode (cluster Type 2), which is associated with large distance movements and relatively large turning angles. Since this is target-oriented movement, the speed in the direction of movement in this pattern is relatively high [58]. Traveling mode is used when an animal is moving from one area to another with an aim, and this is characterized by fewer stops and moves intervals. The final category is the searching or foraging pattern (cluster Type 3), and this is characterized by short-distance movements over a wide geographic area with small turning angles [58]. The speed in the direction of movement in this pattern is low [60] and represents the kind of movement an animal makes when feeding or foraging.

Most of the relocations took place in the afternoon and at night, which indicated that the calves were most active during these times. The least number of relocations were made in the morning. Similarly, the greatest distance was covered in the afternoon and at night. These findings are consistent with a previous study involving lambs, which concluded that animals reduce their movement and spend more time lying down during the hottest times of the day [61]. In response to heat stress, cattle regulate body temperature by reducing movement and feeding [62]. Raizman et al. [63] found that cattle adjusted their visit to a drinking point to once in two days during the dry season instead of daily in the wet season. In our case, the calves may have been trying to minimize their energy expenditure to reduce heat gain and therefore remain lying for longer periods during the day. Our findings suggest a pattern of activity over a 24 h period that is representative of calves coping with mild heat stress.

The low linearity ratio indicated that most of the movements made by the calves in all of the cluster types were turning movements. Cluster type 1 and 2 both recorded the highest turning angles with a mean of −0.2 radians, while cluster type 3 recorded turning angles of −0.03 radians. Since the linearity ratios show that the movement of these calves was characterized more by turning, it is not surprising that the mean angle turns are not significantly different between the three cluster types. This is contrary to previous studies that reported that cluster type 2 (traveling) consists of relatively straight movements [58,59]. Additionally, cluster type 2 recorded the fastest rate of movement (Table 1). This is in agreement with previous findings that type 2 movements have the highest rate of movement [33,58]. It is worth noting some irregularities in movement variables across all identified Clusters where some values include zero measurements. These irregularities could be used as indicators of animal health status (e.g., subclinical or clinical disease). Alternatively, animal movement variables could be have been influenced by the method in which the calves were fed in this study, which may have led to changes in traveling time and travel to different foraging locations. Management factors, such as feeding strategy and frequency, may also influence behaviors [64]. Furthermore, the social interaction between and among the group may affect the behavior of others, as seen in a study to measure gait and feeding behavior in cattle [65,66].

This pilot study has been able to establish a relationship between the four investigated movement variables (distance traveled, ROM, linearity ratio and turning angle). However, due to the small sample size, the validity of these results is limited to the current study and to healthy calves of similar ages, breeds and management factors. Additionally, the combination of both females and males in this cohort has not been evaluated to determine whether this had a role in the movement patterns observed. More research is needed to investigate whether these results are consistent with the movement patterns of other calf herds. Research into how these movement patterns vary during other environmental conditions, such as the different seasons, would also be useful in developing a dataset that could be used to evaluate movement patterns on farms year-round. More work is also needed to determine the feasibility of this technology under field conditions. Finally, there were coordinates that fell outside the boundary of the paddock as the animals were moving close to the boundary. These results are in agreement with the literature [44]; this form of inaccuracy is not unusual with GPS devices as signals can be misdirected from the satellite to the receiver to produce what is commonly referred to as “noises” [44,64]. This is associated with the high dependency of this technology on satellite geometry and weather conditions [44]. These misdirected signals give readings that, if included as part of the data analysis, may give misleading conclusions and, therefore, were removed [67].

## 5. Conclusions

This study described the movement patterns of recently weaned dairy calves and grouped them into three distinct patterns. This study also evaluated the relationship between variables that constitute the three cluster types. There were some irregularities in the movement variables that were used for classifying or characterizing the three cluster type movements as described by previous authors, and these may be due to changes in animal health status, climatic conditions, feeding strategy and the social interactions between the calves. The calves in this study were more active in the afternoon and at night. There was a correlation between the rate of movement, linearity ratio, and the distance traveled. However, turning angles do not have any influence on the distance traveled and the rate of movement across the three cluster types. The findings reported in this study could be used to further develop the interpretation of movement and behavior patterns of calves in order to establish an early detection system for poor health and welfare on dairy farms.

## Figures and Tables

**Table 1:** Summary of overall and cluster-specific movement pattern data for the 11 calves as obtained from each global positioning system (GPS) logger units.

Variable	Overall Movement Pattern		Movement Pattern Cluster						
			One (Resting)		Two (Traveling)		Three (Foraging)		*p*-Value (between Clusters Variance)
	Mean (SD)	Median (min, max)	Mean (SD)	Median (min, max)	Mean (SD)	Median (min, max)	Mean (SD)	Median (min, max)	
Relocations (counts)	1594 (1004)	1288 (618, 3725)	684 (38)	622 (618, 750)	2862 (499)	2862 (1982, 3725)	2275 (300)	2275 (1442, 3050)	<0.01 ^a^
Distance (m)	18 (27)	11 (0.1, 639)	7 (14)	2 (0, 306)	35 (68)	11 (0, 639)	11 (17)	0 (0, 147)	<0.01 ^b^
Rate of movement (m/s)	0.67 (1.10)	0.39 (0.001, 29)	0.30 (0.26)	0.30 (0.00, 2.40)	2.30 (2.80)	1.70 (0.01, 29.00)	0.90 (0.80)	0.90 (0.00, 7.70)	<0.01 ^c^
Linearity ratio	0.002 (0.010)	0.001 (0.001, 0.190)	0.00 (0.00)	0.00 (0.00, 0.01)	0.01 (0.02)	0.01 (0.0, 0.20)	0.00 (0.00)	0.00 (0.00, 0.02)	<0.01 ^d^
Turning angle (radians)	−0.15 (1.80)	−0.02 (−3.14, 3.14)	−0.20 (1.80)	0.04 (−3.10, 3.10)	−0.20 (1.80)	−0.10 (−3.10, 3.10)	−0.03 (1.80)	0.00 (−3.10, 3.10)	0.09 ^e^

Key: SD—standard deviation; min—minimum; max—maximum. ^a^ Chi-squared test derived *p*-value (χ = 1309, df = 2); ^b^ analysis with one-way ANOVA indicated a difference between clusters (F_2, 2682_ = 1277); ^c^ analysis with one-way ANOVA indicated a difference between clusters (F_2, 2682_ = 482); ^d^ analysis with one-way ANOVA indicated a difference between clusters (F_2, 2682_ = 310); ^e^ analysis with one-way ANOVA indicated a difference between clusters (F_2, 2682_ = 2.45).
